# Aspergillus pseudodeflectus: a new human pathogen in liver transplant patients

**DOI:** 10.1186/s12879-018-3527-5

**Published:** 2018-12-12

**Authors:** Nawel Aït-Ammar, Eric Levesque, Jean-Benjamin Murat, Sébastien Imbert, Françoise Foulet, Eric Dannaoui, Françoise Botterel

**Affiliations:** 1 Unité de Parasitologie–Mycologie, Département de Virologie, Bactériologie–Hygiène, Parasitologie–Mycologie, DHU VIC, CHU Henri Mondor, AP-HP, Créteil, France; 2 EA Dynamyc UPEC, ENVA, Faculté de Médecine de Créteil, Créteil, France; 3 Réanimation Digestive et Hépato-biliaire, Service d’Anesthésie et des Réanimations Chirurgicales, CHU Henri Mondor, AP-HP, Créteil, France; 4 Service de Parasitologie-Mycologie, CHU Pitié Salpêtrière, AP-HP, Paris, France; 5 Université Paris–Descartes, Faculté de Médecine, Unité de Parasitologie–Mycologie, Service de Microbiologie, Hôpital Européen Georges Pompidou, AP-HP, Paris, France

**Keywords:** Aspergillosis, Liver transplantation, *Aspergillus pseudodeflectus*, Molecular identification, Azoles resistance

## Abstract

**Background:**

Liver transplant recipients are at high risk of developing invasive aspergillosis and in particular by *Aspergillus fumigatus* which is the most commonly encountered species in this population. Other non-*fumigatus Aspergillus* species with reduced susceptibility to antifungal drugs can also be involved. Accurate identification associated to antifungal susceptibility testing is essential for therapy adjustment. We report a case of invasive pulmonary aspergillosis due to *Aspergillus pseudodeflectus* in a liver transplant recipient. To our knowledge, this is the first reported case of invasive aspergillosis due to this species with a reduced susceptibility to azoles.

**Case presentation:**

A 64 year-old woman with drug-induced fulminant hepatitis underwent liver transplantation. Prophylactic treatment with caspofungin was introduced due to aspergillosis risk factors consisting in hemodialysis and fulminant hepatitis. Six weeks after transplantation, CT scan showed a right pulmonary opacity associated with an increase of galactomannan (index 5.4). Culture of BAL grew with several colonies of *Aspergillus* sp. The diagnosis of invasive aspergillosis was probable according to the EORTC criteria. The antifungal susceptibility tests (Etest®) revealed low MICs to echinocandins and amphotericin B) but high MICs to azoles. After these results, voriconazole was switched to liposomal amphotericin B. The patient died one month after diagnosis from a refractory septic shock with multiple organ failure. A molecular identification of isolate, based on partial β-tubulin and calmodulin genes, was performed and identified *A. pseudodeflectus.*

**Conclusions:**

Our case raises the question of pathogenicity of this species, which belongs to *Aspergillus* section *Usti* and is genetically and morphologically very close to *Aspergillus calidoustus* that was previously reported in human transplant recipients.

## Background

Invasive aspergillosis (IA) has been reported in 1–15% of organ transplant recipients [1–4]. In liver transplant recipients, it is the second most common invasive fungal infection (IFI) after candidiasis with a rate of 1–9.2% [4]. Aspergillosis is usually associated with high mortality (83 to 88%) [1] and is most commonly caused by *Aspergillus fumigatus* [2]. Other non-*fumigatus Aspergillus* species are currently emerging as substantial cause of invasive aspergillosis [5–8]. Thus, identification of the species type appears to be important due to the variable susceptibility of the members of genus *Aspergillus* to antifungal drugs [8–11]. The current taxonomy of *Aspergillus* showed cryptic species, of a single section, which are morphologically indistinguishable [12] yet may have different antifungal susceptibilities with higher minimum inhibitory concentrations (MIC) [9, 12–15]. The phylogenetic relationship between species of *Aspergillus* is analyzed by sequencing β-tubulin and calmodulin encoding genes regions [16].

Cryptic species from section *Usti* such as *A. ustus*, *A. calidoustus,* and *A. granulosus* are reported as an emerging cause of invasive aspergillosis [7, 17–20]. However, *A*. *pseudodeflectus*, another species of section *Usti* [21, 22], has never been reported as a causative agent of human aspergillosis. We report here the first case of invasive pulmonary aspergillosis induced by *A. pseudodeflectus* in a liver transplant recipient.

## Case presentation

A 64-year-old woman known to have cirrhosis secondary to Hepatitis C was transplanted in our center to treat drug-induced fulminant hepatitis failure. The patient received a standard post-op immunosuppressive protocol (including corticosteroids, tacrolimus, and mycophenolate mofetil).

She also received caspofungin (70 mg at day 1 then 50 mg/day) according to ESCMID recommendations as targeted prophylaxis against IA during 15 days [23]. The early post-operative period was associated with hemorrhagic episodes and peri-hepatic hematoma requiring several re-interventions whilst maintaining the antifungal prophylactic treatment. From post-operative day (POD) 31, the patient developed several septic shocks caused by *Enterococcus faecium*, *Escherichia coli*, and *Candida glabrata,* and all were treated by broad spectrum antibiotics and caspofungin was reintroduced.

**Fig. 1:**
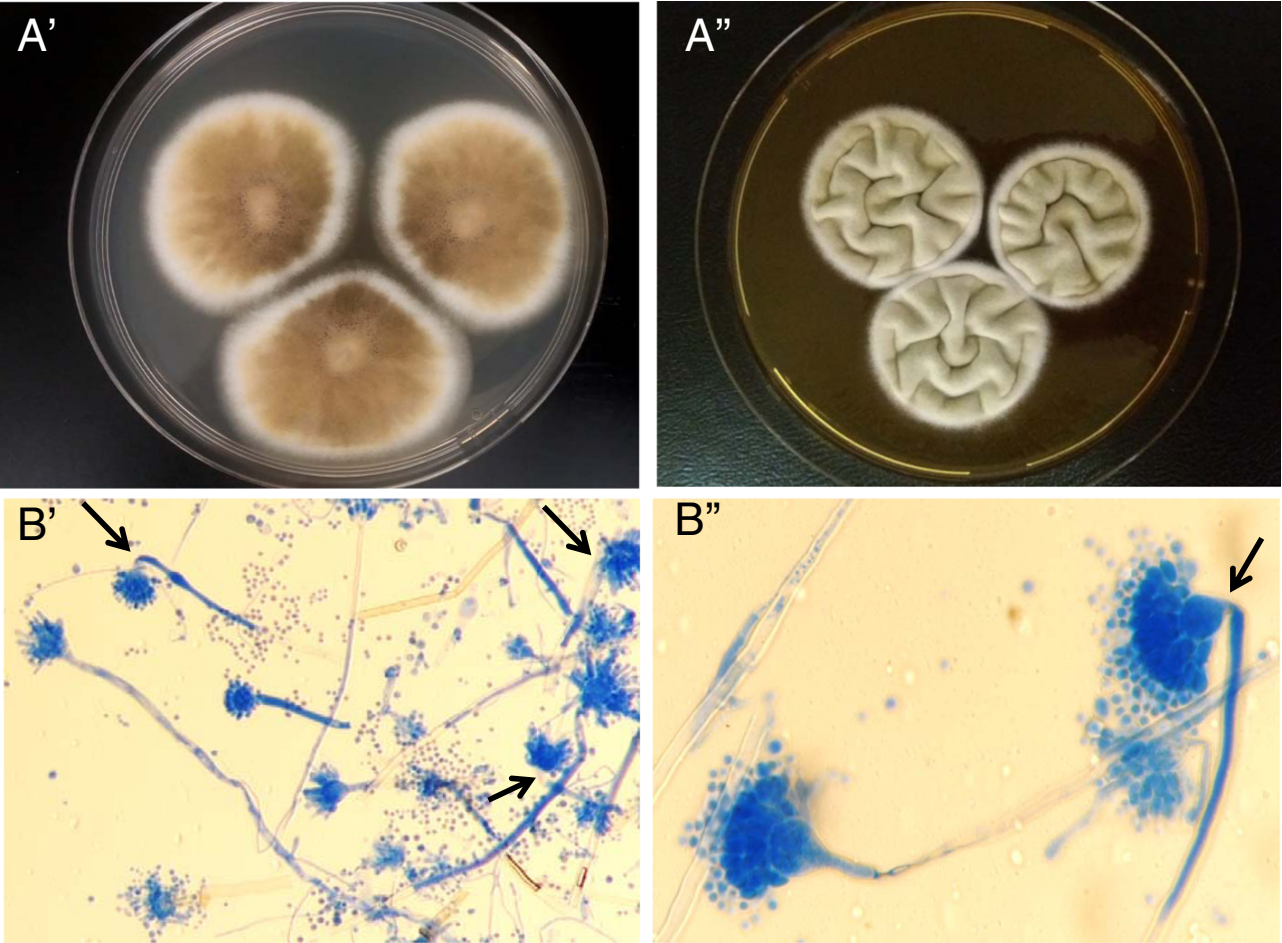
*Aspergillus pseudodeflectus* isolated from BAL. **a**. Colony at 25 °C after 7 days. A’. Malt Extract Agar (MEA). A*"*. Czapek Yeast Autolysate Agar (CYA) + 20% sucrose. B. Microscopic aspect with blue lactophenol staining (CYA). **b**′. Magnification × 200. B″. Magnification × 1000. Arrows show curved conidiophores

On POD 63, the patient got fever resistant to antibiotics. Her chest CT-scan showed right-sided pleural effusion with passive atelectasis and alveolar opacity. Broncho-alveolar lavage (BAL) was performed and its direct examination displayed *Aspergillus*-like branched hyphae. At the same time, galactomannan (GM) antigen index (Platelia Aspergillus, BioRad) and (1–3)-β-D-glucan (BDG) (Cape Code) in serum, which were previously negative, became positive (GM antigen index > 6 (threshold index: 0.5), BDG = 234 pg/mL (threshold value: 80 pg/mL)). *Aspergillus* real-time PCR (qPCR), based on a target of 67-bp DNA fragment specific to the multicopy gene encoding the 28S rRNA of *A. fumigatus*, was positive in serum (Cq value = 35) [24]. Eventually, based upon data from the European Organization for Research and Treatment of Cancer/Mycoses Study Group (EORTC/MSG) [25], the patient was classified as having a probable IFI. Caspofungin was then switched to voriconazole (200 mg twice/day). On Sabouraud media, at 37 °C, the culture of BAL showed growth of several greenish to brownish colonies of filamentous fungi with a powdery aspect. Macroscopic examination revealed brown colonies on Malt media and surrounded by a white mycelium. The reversed side of the colonies was yellow. On Czapek Yeast Autolysated Agar (CYA) media, the detected colonies were much greener in color with velvety texture. Microscopic examination of the colonies showed *Aspergillus* biseriate conidial heads with curved conidiophores (Fig. 1). On the basis of these macroscopic and microscopic examinations, the species can be suggested but must be confirmed by molecular identification. The follow-up GM antigen index, even with voriconazole, remained positive (index> 6 at POD 65 and 70). Antifungal drug susceptibilities were determined by Etest on RPMI medium supplemented with 2% glucose. MICs were read at 48 h of incubation at 35 °C. MICs of amphotericin B, itraconazole, voriconazole, posaconazole, micafungin, and caspofungin were 0.75 μg/mL, 12 μg/mL, 4 μg/mL, 6 μg/mL, 0.016 μg/mL, and 0.5 μg/mL, respectively. Since the antifungal susceptibility revealed elevated MICs to azoles, voriconazole was then switched to liposomal amphotericin B. Additionally the results of EUCAST method confirmed the high MICs to azoles. On POD 81, the patient died from multiple organs failure and refractory septic shock secondary to pneumonia. Autopsy was not performed.

**Fig. 2:**
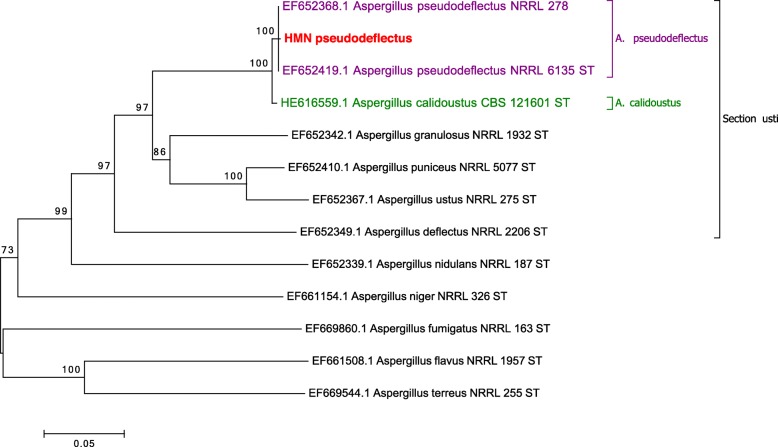
Neighbour-joining tree based on β-tubulin and calmodulin genes sequence data of HMN *Aspergillus pseudodeflectus*. Numbers above the nodes represent bootstrap values. These values were generated from 1000 replicates. The bar length represents the specified genetic distance. Only values above 70% are indicated

A molecular identification of this *Aspergillus* was performed. Complete genomic DNA was extracted from a mature subculture on Sabouraud agar using QIAamp DNA Blood Mini Kit (Qiagen Sciences Ing.) after a step of bead beading in MagNA Lyser Instrument (Roche). The rDNA of partial β-tubulin and calmodulin genes were amplified as described by Samson et al. [16]. Sequencing reactions were carried out for both strands. When compared with the partial calmodulin gene sequences available in the NCBI database, the highest identity was obtained with *A*. *pseudodeflectus* NRRL 278 strain (Genbank accession number EF652368.1) and NRRL 6135 strain (Genbank accession number EF652419.1) [16] with nucleotide identity rates of 100 and 99%, respectively over a sequence length of 573 bp. For the partial β-tubulin sequences, identity rates of 99% were obtained with both strains, NRRL 278 (Genbank accession number EF652280.1) and CBS 596.65 (Genbank accession number EF591732.1). Phylogenetic trees of these two sequences, alone and combined, were built with the MEGA 6.05.1 software [26]. The neighbor-joining method, using the Kimura two-parameter model with 1000 bootstraps replications, was applied to each data set. The sequence relatedness of our strain with the species strains type of section *Usti* is shown in Fig. 2.

## Discussion and conclusion

We report the first case of IA caused by *A. pseudodeflectus.* This species was first described by Samson and Mouhacca in 1975 in Egyptian desert soil samples [27]. *A. pseudodeflectus* belongs to *Aspergillus* section *Usti* [21, 22]. This section includes more than 20 species. *A*. *calidoustus* was isolated from air and water distribution systems in hospital environment, after some clinical cases [28–30]. Taxonomy of this section keeps evolving as two new species have recently been reported [31]. Outbreaks have already been reported, although the infection source was not clearly identified [18, 32] . No data are available on environmental or aerial distribution of *A. pseudodeflectus*. In our patient, inhalation is probably the source of infection yet no air sampling was performed in the surgical department and no other similar case was declared in the same ward.

*Aspergillus* section *Usti* species are rarely pathogenic but can be opportunistic agents. The immunocompromised patients affected by these fungi are hematopoietic stem cells transplant (HSCT), lung transplant, and heart transplant patients [6, 17, 19, 33–37]. Pulmonary and cutaneous infections are the most common complaints [6, 18, 33–35, 37]. GM index can be positive in patients infected by *Aspergillus* species of this section [38] and the clinical evolution and our case confirms these data. *Aspergillus* PCR and BDG were also positive in our patient but no data are available in the literature to validate these diagnostic tools in aspergillosis induced by section *Usti*.

Molecular identification is recommended to avoid false identifications because species within a single section cannot be morphologically [39] distinguished from each other [12] . Previous cases of IA caused by *A. ustus* were misidentified [21], mostly as *A. calidoustus* [40]. The latter was included as a new species in 2008, and ever since it has been the most commonly reported cause of IA in this section [21]. In contrast to *A. ustus,* which cannot grow at 37 °C, *A. pseudodeflectus and A. calidoustus* can grow at 37 °C, and this could explain their human pathogenicity [22, 41]. *A*. *pseudodeflectus* has never been described in human pathology but it has probably been underestimated before the establishment of molecular identification.

*Aspergillus* molecular identification is based on sequencing partial β-tubulin and calmodulin genes [39]. Sequencing of ITS regions does not enable clear differentiation between the species of a single section. Our results (Fig. 2) show that our isolate of *A. pseudodeflectus* is closely related to *A. calidoustus*. Other non-molecular parameters can be used to distinguish *A. pseudodeflectus* from *A*. *calidoustus*, like a negative Ehrlich reaction and curved conidiophores, as described in our case [41] .

To our knowledge, there are few data on antifungal susceptibilities of section *Usti*. *A. calidoustus* has been reported as intrinsically resistant to azoles [8, 14, 21, 42–44], and this imposes management difficulty since voriconazole is the first line reference treatment of aspergillosis [45]. Echinocandins and amphotericin B MICs seem to be more variable [21, 42]. Antifungal susceptibilities of *A. pseudodeflectus* are not well known but the MICs calculated on our isolate suggest that it is resistant to azoles like the other species of complex *Usti*. Although, echinocandins and amphotericin B MICs of our isolate were relatively low, our patient developed aspergillosis while being on caspofungin prophylaxis treatment. The treatment was switched first to voriconazole and then to liposomal amphotericin B but the patient did not recover. In a review on IA due to *A. calidoustus* in HSCT patients treated with liposomal amphotericin B, a poor outcome was also observed [18]. This suggests that Amphotericin B may not be very effective. Interestingly, terbinafine showed low MIC to *A. calidoustus* [43] but this treatment is difficult to manage in immunocompromised and critical care patients.

Our case reports an invasive pulmonary aspergillosis in a liver transplant recipient caused by *A*. *pseudodeflectus,* a species that is newly described in human diseases and presented resistant to azoles. The use of molecular tests is important for *Aspergillus* identification because of its cryptic species and potential antifungal resistance which cannot be detected by routine techniques. Other studies on epidemiology, diagnostic tools, and antifungal susceptibility of *Aspergillus* section *Usti* are needed.

## Abbreviations

BAL: Broncho-alveolar lavage
BDG: (1–3)-β-D-glucan
CYA: Czapek Yeast Autolysated Agar
GM: Galactomannan
HSCT: Hematological stem cells transplantation
IA: Invasive aspergillosis
IFI: Invasive fungal infection
MIC: Minimum inhibitory concentrations
POD: Post-operative day
